# Earthworm species and density in semi-natural grasslands on rice paddy levees in Japanese *satoyama*

**DOI:** 10.3897/BDJ.8.e56531

**Published:** 2020-09-29

**Authors:** Keiko Kishimoto-Yamada, Yukio Minamiya

**Affiliations:** 1 Sado Island Center for Ecological Sustainability, Niigata University, 1101-1 Niibo-katagami, Sado, Niigata 952-0103, Japan Sado Island Center for Ecological Sustainability, Niigata University 1101-1 Niibo-katagami, Sado, Niigata 952-0103 Japan; 2 Tochigi Prefectural Museum, 2-2 Mutsumi-cho, Utsunomiya, Tochigi 320-0865, Japan Tochigi Prefectural Museum 2-2 Mutsumi-cho, Utsunomiya, Tochigi 320-0865 Japan

**Keywords:** agricultural landscapes, Haplotaxida, Lumbricidae, Megascolecidae, Oligochaeta, preys, Sado Island

## Abstract

Earthworms contribute to the sustainability of food webs in the semi-natural grasslands of levees at paddy margins, which are typical components of *satoyama*, the traditional agricultural landscapes of Japan. Thus far, few studies have focused on earthworm fauna of paddy levees in *satoyama*. In this study, we investigated earthworm fauna and regional and monthly changes in earthworm density. We found at least 11 species of earthworms living within levees on Sado Island, central Japan. Two endogeic species, *Amynthas corticis* (Megascolecidae) and *Eisenia japonica* (Lumbricidae), were dominant in terms of number of adult individuals; these two species appeared in all study regions. We also estimated an average of ~57.4 individuals/m^2^ for all stages of earthworms in levees, suggesting that rice paddy levees have relatively-high earthworm density. However, such tendencies differed depending on the region. In addition, monthly changes in density were observed in the topsoil of the levees. Our results imply that differences in earthworm assemblages amongst regions and months may influence the availability of food resources for various animals inhabiting *satoyama*.

## Introduction

Terrestrial earthworms are a high-potential food resource for secondary consumers (e.g. Lee 1985, Onrust and Piersma 2017). In *satoyama*, traditional agricultural landscapes of Japan, various types of animals [e.g. Japanese marten (Tatara and Doi 1994), moles (Yokohata 2005), birds (Minamiya et al. 2007, Nagata 2010, Endo and Nagata 2013), frogs (Hirai and Matsui 2000, Hirai and Matsui 2001) and carabid beetles (Okuzaki et al. 2010)] feed on earthworms. Thus, earthworms contribute to the sustainability of food webs in *satoyama*.

The levees of rice paddies, located at paddy margins and separating paddies from secondary forests, are typical components of *satoyama*. They are constructed and maintained to retain water in the paddies and to allow the passage of people and transportation of tools (Matsumura et al. 2014). Farmers generally maintain levee grasslands by periodic mowing or herbicide application. As large semi-natural grasslands have been converted to other land uses in Japanese *satoyama*, grasslands on rice paddy levees have recently been re-discovered as important refuges for grassland species of plants and animals. For example, previous studies demonstrated that traditional management practices for levee grasslands maintain high diversities of herbivore and plant species, including rare plant species (Uematsu and Ushimaru 2013, Uchida and Ushimaru 2014, Ohara and Ushimaru 2015). However, most studies of biodiversity in levee grasslands have focused on plant species; few have examinedthe soil fauna of paddy levees.

On Sado Island, central Japan, typical *satoyama* landscapes occur throughout the island. In addition, a re-introduction programme for the once-extinct Asian crested ibis, *Nipponia nippon*, has been underway on the Island since 2003 (Nagata and Yamagishi 2016). This bird species is listed as Endangered on the IUCN Red List 2018. Since re-introduction, the wild population of crested ibis has been steadily increasing; the recent population estimate is 368 wild individuals in 2018 (Bansho 2019). According to a recent study, wild crested ibis feed on earthworms on the levees of rice paddy margins, especially in the summer (Endo and Nagata 2013). However, the species composition and density of earthworms within levee grasslands on the island have not been examined. Basic information regarding earthworm fauna in semi-natural grasslands of rice paddy levees is necessary for efforts, such as the development of habitat management strategies and maintenance of viable crested ibis populations, as well as the conservation of foraging habitats of various animals living in *satoyama*. Therefore, the objective of the present study was to examine earthworm fauna, as well as regional and monthly changes in earthworm density on Sado Island.

## Materials and methods

### Study site

**Figure 1.**
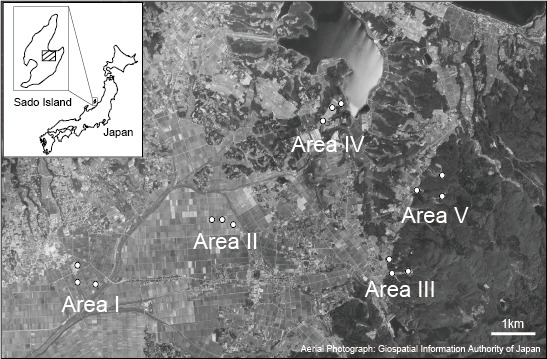
Satellite image of the study site.

This study was conducted on Sado Island, located in the Sea of Japan, approximately 40 km off the coast of Niigata Prefecture (138°E, 38°N; Fig. 1). The Island has an area of 855.25 km^2^; it consists of two mountainous areas (Osado and Kosado) separated by the Kuninaka Plain. Rice fields cover almost the entire Kuninaka Plain. In Osado and Kosado, rural communities with rice fields are dotted along the mountains and coastline.

Five study regions on the Kuninaka Plain and in Kosado were selected (Fig. 1). These regions had different surrounding landscapes and farmland consolidation conditions. Areas I and II were surrounded by cultivated lands, while Areas III–V were surrounded by forests (Fig. 1, Table 1). In this study, farmland consolidation was considered to mean that the soil of the levee had been replaced once. More than 10 (13–37) years had passed since the completion of paddy consolidation in Areas II, IV and V (Table 1). In each region, three rice paddies, separated by at least 200 m, were selected. The areas of the study paddies were 399–4,983 m^2^ (Table 1). In the studied paddies, farmers managed grasses on the levees by mowing two to five times per year and no herbicide was used. After mowing, litter was usually left on the levees; however, some farmers swept the litter away. Various grass species were found on the levees, such as *Equisetum arvense*, several *Rumex* and *Trifolium* species, *Zoysia japonica* and several other Poaceae and Polygonaceae species.

### Earthworm sampling

Sampling was conducted monthly from June to September 2017, which is the irrigation season. On Sado Island, earthworms were frequently fed by the wild crested ibis on paddy levees during this period (Endo and Nagata 2013) and a previous study revealed that earthworm abundances decreased by half and several species disappeared as of autumn (Harada and Yoshimura 2015). Three points on two or three levees surrounding each rice paddy were selected. At each point, a soil monolith (25 × 50 cm, 10 cm deep) was taken. Earthworms found in each monolith were hand sorted and all individuals with and without clitellum were counted in the field. The numbers of all individuals collected from all three points per rice paddy and per month were added. Only individuals with clitellum (i.e. adults) were taken to the laboratory, fixed with a 10% formaldehyde solution and preserved in 80% ethanol for species identification. The specimens are preserved at Tochigi Prefectural Museum (TPM).

### Data analysis

To examine regional and monthly changes in earthworm densities, the densities were compared amongst study locations using the Kruskal-Wallis test and amongst months using the Friedman test, followed by post hoc analysis. Post hoc tests were conducted using the Nemenyi test in the PMCMR package. All analyses were performed using R version 3.3.3 (R Core Team 2017).

## Data resources

Species occurrence data of levee earthworms collected on Sado Island are listed in Suppl. material 2.

## Results

The examination of individuals with clitella revealed the presence of eight Megascolecidae species, two Lumbricidae species and one Moniligastridae species at the levees (Table 2). *Amynthas corticis* and *Eisenia japonica* were the dominant species in terms of the number of adult individuals and both species appeared at all study regions (Table 2). These two species comprised approximately 85% of all adult individuals (N = 192); all other species only appeared infrequently (Table 2). Four species, *A. aokii*, *A. micronarius*, *Metaphire agrestis* and an unidentified species of Moniligastridae, appeared only at non-consolidated levees (Areas I and III).

The ten species identified in this study can be categorised into two functional groups (Uchida and Kaneko 2004, Ishizuka 2015): five epigeic and five endogeic species (Table 2). Endogeic species comprised approximately 91% of all adult individuals (N = 190, with the exception of the unidentified species of Moniligastridae).

**Figure 2.**
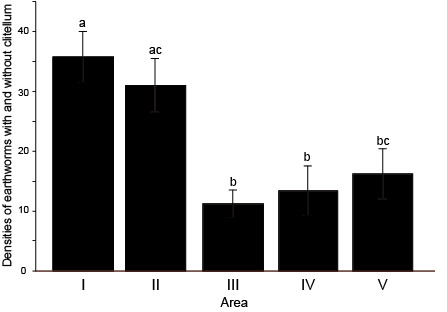
Comparison of densities of earthworms with and without clitellum amongst study regions. Different letters indicate statistically-significant differences (p < 0.05).

During the study period, 1,292 individuals with and without clitella were collected. The mean density was 21.53 (0–58) individuals/0.375 m^2^. Amongst the five regions, densities tended to be different (Kruskal-Wallis chi-squared = 22.53, p < 0.001): they were high in the Areas I and II, compared to Areas III–V (Fig. 2).

**Figure 3.**
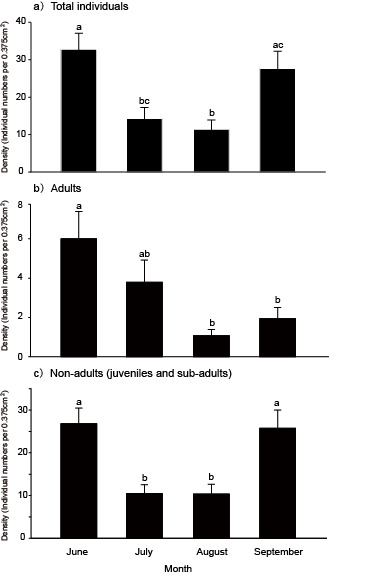
Comparison of densities of a) total individuals, b) adults with clitellum and c) non-adults without clitellum amongst months. Different letters indicate statistically-significant differences (p < 0.01).

In addition, the total numbers of individuals tended to be different (Friedman chi-squared = 29.98, p < 0.001): they were low in July and August, compared to values observed in June and September (Fig. 3a). Adults (individuals with a clitella) comprised 7% of all individuals and they also tended to be different (Friedman chi-squared = 22.49, p < 0.001), with adult density peaking in June and then decreasing in August and September (Fig. 3b). The densities of non-adult individuals (no clitella) were different (Friedman chi-squared = 22.49, p < 0.001): they were low in July and August, then recovered in September to densities comparable with those observed in June (Fig. 3c).

## Discussion

This study identified at least 11 species of earthworms living within levees on Sado Island. Previously, only seven species (*Amynthas divergens*, *A. hupeiensis*, *Metaphire acincta*, *Aporrectodea caliginosa*, *E. japonica*, *Helodrilus hachiojii* and *Drawida hattamimizu*) had been recorded at rice paddy levees in Japan (Blakemore et al. 2010, Kiritani 2010). Eight species, including an unidentified Moniligastridae species, were newly observed in this study.

Species diversity and evenness tended to be low at the rice paddy levees; only two species, *A. corticis* and *E. japonica*, were dominant. *E. japonica* is present in most ecosystems in Japan, including mountainous forests and grasslands (Hokkaido Development 1965). Kamihira (1973) reported that this species is found more frequently in grasslands than on forest floors; it also inhabits farmland and residential areas. *A. corticis* is often observed under decayed trees, in landfills and in parks, amongst other locations (Reynolds 1978). Farmers often walk upon, transport tools and periodically cut the grasses on rice paddy levees, particularly during the irrigation season. The two dominant earthworm species are presumably able to tolerate such habitats that experience relatively-intensive disturbances.

Levee grasslands may also be characterised by relatively-high earthworm densities. For example, previous comprehensive studies conducted in pastures and meadows in Hokkaido, northern Japan, recorded 35.5–200.4 individuals and 14.9–49.7 g/m^2^ biomass (Nakamura 1967, Kamihira 1968, Kamihira 1969). We observed averages of approximately 57.4 individuals/m^2^ at the levees. The area of each paddy levee is generally very small. In addition, compared with other semi-natural grasslands, such as pastures and meadows, disturbance frequencies tend to be higher on levees, in terms of management by farmers (Ushimaru 2012). Nevertheless, the values obtained in this study were nearly within the ranges observed in pastures and meadows. Thus, we regard these semi-natural grasslands, which are typical components of *satoyama* landscapes, as important habitats for earthworms and their predators.

However, in the present study, we observed that earthworm densities differed amongst regions. In particular, densities were high at the levees without adjacent forest, compared to levees with adjacent forests. The situation of farmland consolidation and characteristics in soil properties (Soil pH, C, N and C:N ratio) is unlikely to differ between these two types of levees (Table 1). Structures at margins between paddies and forests may reduce earthworm densities. Sklenicka et al. (2002) reported low porosity and infiltration rates in the soil in crop fields near forest edges and suggested that the phenomenon was caused by greater soil compaction, because the field boundaries are often used as corridors for agricultural machinery traffic. Alternatively, earthworm densities in levee grasslands with adjacent to forests may also be negatively affected by increased predation at the forest edge. For example, Andren (1992) found that the densities of corvid birds tended to increase in fragmented forests, as well as at the boundaries between forests and agricultural lands. Positive edge effects have also been documented for carabid beetles in various regions (e.g. Molnár et al. 2001, Magura 2002), although the effects differ amongst seasons (Ohwaki et al. 2015). In addition, the number of frog egg masses in paddies has been positively correlated with high proportions of surrounding forests within a 1000-m radius on Sado Island (Uruma et al. 2012). These animals often feed on earthworms; therefore, high predation pressure at forest edges may reduce earthworm densities. Further investigations are needed to determine why earthworm density differs amongst different landscapes, particularly those with and without adjacent forest.

We also observed that epigeic species were rarely found at the levee grasslands in this study. Epigeic species live in the litter layer and feed on litter (Uchida and Kaneko 2004, Ishizuka 2015). The unstable supply of litter on levees may be a factor in their low density. Two types of litter were found on the paddy levees: grass and forest litter. Both litter types are not likely to be permitted to decay, thus becoming palatable for earthworms; litter is often removed by passing farmers and machinery. In addition, the levees are usually open and litter tends to be blown away by the wind; farmers may also sweep the litter from levees after mowing. Thus, presumably due to the unstable supply of litter, epigeic species appear to be unable to settle on the levees.

Monthly changes in earthworm densities occurred in the topsoil of the levees. The changes are likely linked to the life cycles of some species. For example, for some endogeic species of Megascolecidae, cocoons hatch from April to September; thus, juveniles and adults appear throughout the year (Ishizuka 2015). Several species found in this study may exhibit this type of life cycle, as we measured high densities of non-adult individuals in September; all stages were found throughout the study period. However, in terms of *E. japonica*, the dominant species in this study, the monthly changes in density presumably did not reflect its life cycle. For example, Nakamura (1972) showed that the life cycle of *E. japonica* is such that adults appear mainly from May to October; they peak in number in August. In contrast, we observed that adults rarely appeared in August and then disappeared from the 0–10-cm layer in September. At least for this species, monthly changes in earthworm density could reflect movement from the topsoil to deeper layers. A previous study has demonstrated that the proportions of *E. japonica* adults and sub-adults in the 10–20-cm layer are high in August; earthworms were found mostly in the 0–10-cm layer in other months (Nakamura 1972). More detailed investigations are necessary to better understand the mechanisms driving monthly changes in earthworm density within shallow soil layers. At the very least, such monthly changes are likely to influence the availability of food resources for earthworm feeders on paddy levees. On Sado Island, re-introduced crested ibis feed primarily on earthworms in paddy levees during the period of late July to September (Endo and Nagata 2013). In addition, they cannot dig to depths deeper than 10 cm. Their food resources may be insufficient in July and August. Thus, to conserve the foraging habitats of the crested ibis and various other animals living in *satoyama*, consistent monitoring of earthworms at the levees is necessary, combined with further studies of the mechanisms underlying monthly and regional changes.

## Supplementary Material

7B5035D7-4271-5797-A280-F8B8B56D61C3 10.3897/BDJ.8.e56531.suppl1 Supplementary material 1 Methods for the measurements of landscape conditions and soil properties Data type methods File: oo_447938.docx https://binary.pensoft.net/file/447938 Keiko Kishimoto-Yamada and Yukio Minamiya

8F0E1F7E-1A0D-54FA-B66C-D93BCB53FE3B 10.3897/BDJ.8.e56531.suppl2 Supplementary material 2 Species occurrences of levee earthworms on Sado Island. Data type occurrences File: oo_431319.xlsx https://binary.pensoft.net/file/431319 Keiko Kishimoto-Yamada and Yukio Minamiya

## Figures and Tables

**Table 1.** Detailed characterisations for each study paddy and region. See Suppl. material 1 for the methods for the measurements of landscape conditions and soil properties.

Study Region	Paddy	Area of paddy (m^2^)	Farmland consolidation	Mean values of the percentages of each land-cover type (%) within a 100-m radius			Soil properties			
				Cultivated land	Deciduous broad- leaved forests	Planted forests	pH (H_2_O)	Carbon %	Nitrogen %	CN ratios
I	1	1,023		100.0	0.0	0.0	5.4	3.62	0.33	10.87
	2	613	none	80.2	0.0	0.0	5.4	3.13	0.3	10.43
	3	1,023		100.0	0.0	0.0	5.5	4.09	0.4	10.26
II	1	3,095		100.0	0.0	0.0	5.5	2.59	0.24	10.67
	2	3,855	done	100.0	0.0	0.0	5.7	2.68	0.26	10.14
	3	4,983		100.0	0.0	0.0	5.6	4.64	0.43	10.77
III	1	762		39.9	4.6	55.7	5.3	3.01	0.26	11.49
	2	968	none	54.7	4.5	39.6	5.5	3.08	0.28	10.83
	3	2,980		66.9	0.0	32.7	5.5	5.19	0.41	12.6
IV	1	2,848		63.9	18.7	0.0	4.9	3.26	0.25	13.3
	2	2,537	done	81.9	0.0	18.3	5.3	4.15	0.33	12.45
	3	1,047		61.5	25.5	0.0	5.3	3.18	0.24	13.51
V	1	399		39.0	16.6	19.7	5.2	2.81	0.22	12.74
	2	847	done	25.6	74.8	0.0	5.5	2.17	0.22	10.02
	3	469		23.8	58.9	15.6	5.2	3.15	0.29	10.79

**Table 2.** Functional groups, monthly individual numbers and regional occurrences of adult earthworm species. En: Endogeic species, Ep: Epigeic species

Earthworm species		Functional groups	Month				Area				
			Jun	Jul	Aug	Sep	I	II	III	IV	V
Megascolecidae											
	*Amynthas corticis* (Kinberg, 1867)	En	39	8	6	24	+	+	+	+	+
	*Amynthas aokii* (Ishizuka, 1999)	Ep	0	1	0	0			+		
	*Amynthas micronarius* (Goto & Hatai, 1898)	En	2	2	0	0	+		+		
	*Amynthas tokioensis* (Beddard, 1892)	Ep	0	4	3	3	+	+	+		+
	*Amynthas vittatus* (Goto & Hatai, 1898)	Ep	0	0	1	0		+			
	*Metaphire acincta* (Goto & Hatai, 1899)	En	2	1	0	0		+	+		
	*Metaphire agrestis* (Goto & Hatai, 1899)	Ep	0	0	2	0			+		+
	*Metaphire hilgendorfi* (Michaelsen, 1892)	Ep	0	1	0	2		+			+
Lumbricidae											
	*Aporrectodea trapezoides* (Dugès, 1828)	En	2	0	0	0	+				
	*Eisenia japonica* (Michaelsen, 1892)	En	45	39	3	0	+	+	+	+	+
Moniligastridae											
	Moniligastridae sp. 1	-	0	1	1	0	+		+		
