# Supplementary material for: Earthworm species and density in semi-natural grasslands on rice paddy levees in Japanese satoyama
Source: Biodivers Data J. 2020 Sep 29;8:e56531. doi: 10.3897/BDJ.8.e56531 (PMC7538448; doi:10.3897/BDJ.8.e56531)
Supplement: Supplementary material 1 — Methods for the measurements of landscape conditions and soil properties [file bdj-08-e56531-s001.docx]

**Methods for the measurements of landscape conditions and soil properties**

Mean values of the percentages of each land-cover type (%) within a 100-m radius of each rice paddy field, measured using vegetation data obtained from a GIS vegetation map (National Survey on the Natural Environment, 6-7th Survey; Ministry of the Environment).

For the measurement of pH (H_2_O), total C and N concentrations, and stable C and N isotope ratios, soil samples were collected from each levee using a 100-ml soil core sampler on April 11–13 2018, and passed through a 2-mm sieve to remove stones and visible roots. Soil samples (10 g) were air dried, mixed with 25 ml water using a shaker, incubated at room temperature for 1 h and used to measure pH (H_2_O) with a pH meter (LAQUAtwin pH-11B; Horiba, Kyoto, Japan). The remaining soil samples were oven dried (60°C) for 48 h, then homogenized with a mortar and pestle and used for analysis of total C and N concentrations and stable C and N isotope ratios using a mass spectrometer (Delta V Advantage; Thermo Fisher Scientific, Waltham, MA, USA) coupled with an elemental analyzer (Flash 2000; Thermo Fisher Scientific).
